# From ACTH-Dependent to ACTH-Independent Cushing's Syndrome from a Malignant Mixed Corticomedullary Adrenal Tumor: Potential Role of Embryonic Stem Cells

**DOI:** 10.1155/2020/4768281

**Published:** 2020-05-04

**Authors:** Claudia Ramírez-Rentería, Ana Laura Espinosa-De-Los-Monteros, Espinosa-Cardenas Etual, Daniel Marrero-Rodríguez, Guillermo Castellanos, Rocio Arreola-Rosales, Guillermo Montoya-Martinez, Keiko Taniguchi-Ponciano, Paola Briseño-Díaz, Mayte Lizeth Padilla-Cristerna, María del Pilar Figueroa-Corona, Mercado Moises

**Affiliations:** ^1^Endocrinology Service, UIM Enfermedades Endocrinas, Hospital de Especialidades, Centro Médico Nacional, S.XXI, Instituto Mexicano del Seguro Social, Av Cuauhtemoc 330, Col. Doctores, CP 06720, Mexico City, Mexico; ^2^UIM Enfermedades Endocrinas, Hospital de Especialidades, 23 Centro Médico Nacional, S.XXI, Instituto Mexicano del Seguro Social, Mexico City, Mexico; ^3^Molecular Biomedicine Department, Center for Research and Advanced Studies, Instituto Politécnico Nacional, Av Cuauhtemoc 330, Col. Doctores, CP 06720, Mexico City, Mexico; ^4^Pathology Department, Hospital de Especialidades, Centro Médico Nacional, S.XXI, Instituto Mexicano del Seguro Social, City. Av Cuauhtemoc 330, Col. Doctores, CP 06720, Mexico City, Mexico; ^5^Urology Department, Hospital de Especialidades, Centro Médico Nacional, S.XXI, Instituto Mexicano del Seguro Social, Av Cuauhtémoc 330, Col. Doctores, CP 06720, Mexico City, Mexico; ^6^Laboratorio de Neuroendocrinología Comparada, Departamento de Ecología y Recursos Naturales, Biología, Facultad de Ciencias, Universidad Nacional Autónoma de México, Exterior Circuit S / N, C.U. Coyoacán 04510, Mexico City, Mexico; ^7^Departamento de Biomedicina Molecular Centro de Investigacion y 49 Estudios Avanzados, Av Instituto Politécnico Nacional 2508, San Pedro Zacatenco, Gustavo A. Madero, 07360 Ciudad de México, Mexico City, Mexico

## Abstract

**Objective:**

To report the immunohistochemical and molecular evaluation of a patient with ectopic ACTH syndrome (EAS) from a MCAT which has single cells with features of both 96 medullary and cortical differentiation. *Case Description and Methods*. A 16-year-old woman presented with severe EAS and a large right MCAT composed of ACTH-secreting cells resembling pheochromocytoma and another lineage similar to adrenal carcinoma. Immunohistochemistry (IHC) showed positivity for medullary (ACTH, chromogranin A, synaptophysin, and PS-100) and epithelial components (inhibin, melan-A, and calretinin). Embryonic stem cell markers were evaluated using RT/PCR and immunofluorescence. After initial surgery, the tumor recurred shifting to rapidly progressive ACTH-independent liver metastasis.

**Results:**

Histopathology and IHC revealed two distinct and intermingled cellular patterns, while some cells immunostained for both medullary and cortical markers. Demonstration of all stem cell biomarkers by RT/PCR and immunofluorescence was predominantly localized to the nucleus, whereas SOX2 immunoreactivity was evident in the cytoplasm as well.

**Conclusion:**

The expression of cancer stem cell biomarkers points towards the involvement of primitive embryonic cells as the origin of this neoplasm and maybe to the clinically aggressive and biochemically changing behavior.

## 1. Introduction

Mixed corticomedullary adrenal tumors (MCATs) are very rare and present as a single heterogeneous mass composed of intimately admixed adrenocortical and medullary cells [1–9]. The medullary component usually consists of pheochromocytes, whereas the cortical component is usually an adrenocortical adenoma or less frequently an adrenocortical carcinoma [1–9]. Clinical and/or biochemical evidence of cortisol hypersecretion, putatively by the cortical component, has been documented in about half of the reported cases of mixed corticomedullary adrenal tumors [1–9]. These tumors are especially rare in young patients. On the contrary, catecholamine hypersecretion by the pheochromocytoma component appears to be less frequent [1–9]. A significant proportion of MCAT is clinically and biochemically nonfunctioning lesions. Although pheochromocytomas have been reported to be the source of ectopic ACTH secretion (EAS) [10], there are no published cases where EAS has been demonstrated in the context of a MCAT. Also, most EAS-associated tumors tend to be well-differentiated tumors that can be treated with surgery and/or coadjuvant therapies over several years, while in mixed tumors, the most aggressive pathology usually determines prognosis. Persistence or recurrence of MCAT has not been extensively studied due to lack of complete biological and clinical testing on most of these tumors due to lack of awareness and multidisciplinary teams. Ectopic sources of hormones may be silent before the tumor reaches a critical mass or if treatments are controlling hormonal secretion, but we are unaware of other cases where a tumor shifts from EAS primary tumor to ACTH-independent metastasis. We herein describe the case of a young woman with ectopic ACTH syndrome due to a MCAT with an unusual biological behavior once it became metastatic.

## 2. Case Summary

A previously healthy 16-year-old woman presented with a 4-month history of progressive 12 kg weight gain, fatigue, and muscle weakness to the point of being mostly bedridden and incapable of walking without assistance. Her menstrual periods had been absent for almost a year, and just two months prior to consultation, she developed easy bruising, facial plethora, acne, and hirsutism, as well as severe depression. The patient is a high school student who does not smoke, consume alcohol, or use any recreational drugs. Her family history is unremarkable, negative for diabetes, thyroid conditions, and cancer. On physical exam, her weight was 77 Kg, height 1.48 (BMI 35.1 Kg/m^2^), blood pressure 150/100 mmHg, pulse 95 and regular, and temperature 37°C. She had marked facial plethora with moon facies, truncal obesity, and severe muscle atrophy in upper and lower extremities, as well as inflammatory acne and terminal hair growth over her chin, arms, and chest (Ferriman–Gallwey score of 8). Her abdomen, upper arms, and thighs were covered by multiple purple striae up to 5 cm wide and 20 cm long.

**Figure 1:**
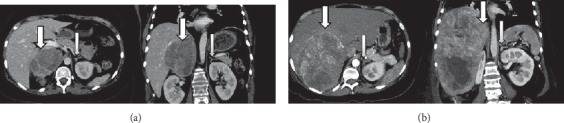
High-resolution, contrast abdominal CT scan, at diagnosis (a) and 16 months after right adrenalectomy (b). (a) Axial and coronal views showing a large, heterogeneous right adrenal mass and an atrophic left adrenal gland. (b)Axial and coronal views showing a large, heterogeneous hepatic mass and a somewhat hypoplastic left adrenal gland.

General laboratory evaluation revealed a serum glucose of 100 mg/dL (normal 70–99), mild hyponatremia of 134 mEq/L (normal 135 a 145), and hypokalemia of 3.2 mEq/L (normal 3.5–5.0) and a normal corrected calcium with mild hypoalbuminemia, normal renal function tests, and mildly elevated liver enzymes. The cell blood count revealed relative lymphopenia and normocytic anemia. Hormonal work up showed normal thyroid function tests, a low-normal estradiol level with suppressed gonadotropins and a serum total testosterone of 42 *μ*g/dL (normal < 40 in females), and dehydroepiandrosterone sulphate (DHEA-S) of 379 *μ*g/dL (normal 61–493). Morning serum cortisol was 43 *μ*g/dL (normal 4.5–24) suppressing to 38 *μ*g/dL after 1 mg of dexamethasone administered the night before (normal suppression < 1.8); urinary free cortisol (UFC) was 2798 *μ*g/day (normal 12.8–82.5), and plasma corticotrophin (ACTH) 36 pg/mL (normal 10–50, ACTH >15 in the context of Cushing's syndrome is considered to be ACTH dependent and <5 is ACTH independent). Her serum cortisol was basically unchanged after an 8 mg, overnight dexamethasone suppression test. Inferior petrosal sinus venous sampling revealed a central-to-peripheral ACTH gradient of 1.3 after the administration of desmopressin. Other hormonal tests included a calcitonin <2 pg/mL (normal < 5) and a chromogranin A of 19 pg/mL (normal < 15), as well as normal serum and urinary catecholamines and 5-hydroxiindolacetic acid. Pituitary magnetic resonance imaging (MRI) was normal, but a thoracoabdominal, high-resolution, contrast CT scan disclosed a heterogeneous, mostly hypodense, 9.3 × 7.6 × 10.4 cm lesion arising from the right adrenal gland (27–61 HU); the left adrenal gland looked atrophied (Figure 1(a)). Metaiodobenzylguanidine (MIBG) scintigraphy did not show any adrenal uptake. A 99Tc-HYNIC scan revealed mild uptake in the right adrenal gland.

**Figure 2:**
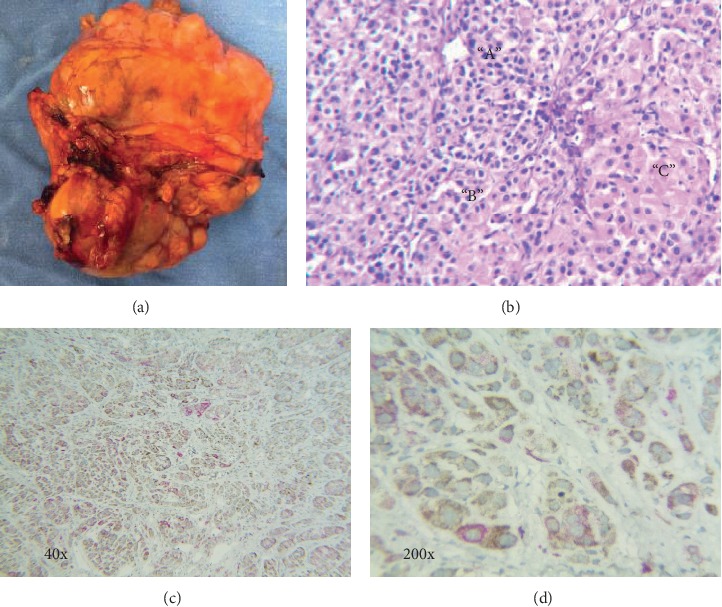
(a) Macroscopic view of the excised tumor. (b) Hematoxylin and eosin stain of the tumor (10x) showing three cellular patterns: “A” corresponds to the medullary neuroendocrine component, “C” corresponds to the cortical component, and “B” shows cells of an intermediate phenotype. (c, d) Double-label immunohistochemistry, with antibodies to CgA linked to horseradish peroxidase (brown) and antibodies against inhibin linked to alkaline phosphatase (red).

She was started on spironolactone, losartan, and amlodipine for blood pressure control, as well as potassium replacement. Two days after admission, she developed hyperglycemia with a fasting serum glucose level up to 650 mg/dL that was treated with an infusion of regular insulin. She underwent open resection of the right adrenal mass without complications. Postoperatively, she presented clinical and biochemical hypocortisolism, with serum cortisol levels below 2 *μ*g/dL (normal 5–20). Over the ensuing few weeks, her clinical condition improved remarkably, losing 15 Kg, serum potassium remained normal without the need for specific replacement, and she began walking without assistance. She was discharged home with normal blood pressure, serum potassium, and serum glucose. Since we have no mitotane available, she completed 31 sessions of external beam radiotherapy to a total dose of 55.8 Gy. She was able to come off glucocorticoid replacement 5 months after the surgical removal of the adrenal tumor, and her UFC remained normal. Three months after surgery, control imaging and laboratory tests did not show any tumor activity. Tumor pathology showed a mixed adrenal pathology with a Ki-67 of 40%. All details on microscopy and molecular findings are detailed in the methods section. Sixteen months after surgery, her UFC started to increase again. Symptoms, signs, and comorbidities of hypercortisolism eventually reappeared, including weight gain, easy bruising, a few purple striae, hair loss, and fatigue, and she developed fasting hyperglycemia. At this point, her UFC rose to 849 mcg/day, but her plasma ACTH remained suppressed (2.35 pg/mL), and therefore, endogenous hypercortisolism was again diagnosed but in this occasion, considered to be ACTH-independent; the testosterone level rose to 88.5 ng/dL, but her DHEA-S remained within normal limits (normal ranges and laboratory history in Table 1). This change in the biochemical and clinical behavior of the tumor prompted additional molecular and medical tests. A control CT scan of her abdomen revealed two large, metastatic liver lesions, measuring 6.4 and 8 cm, respectively, as well as a 7.6 cm peritoneal implant (Figure 2(b)). She declined biopsy of the liver metastases. She was started on palliative chemotherapy with paclitaxel and carboplatinum, without any biochemical or clinical response. The patient stopped visiting the hospital 4 months after the initial chemotherapy due to symptom progression and deteriorating functional status.

## 3. Methods

The patient and her legal guardian signed the appropriate informed consent and confidentiality agreement for the case review and publication after careful explanations. All investigations were performed on the excised tumoral tissues as well as on peripheral blood and urine samples obtained before and after surgery.

### 3.1. Endocrine Evaluation

Hormonal determinations were performed using commercially available immunoassays as follows: serum cortisol was measured by means of the Liaison assay (REF 313261, DiaSorin, Italy), with a sensibility of 0.16 *μ*g/dL, and inter- and intra-assay coefficients of variation (CV) of 4.8% and 5.0%, respectively. For urine cortisol, we used the same immunoassay after methylene chloride extraction. ACTH was measured by a chemiluminescent immunoassay (REF 313221, DiaSorin, Italy) with a detection limit of 1.6 pg/mL and inter- and intra-assay CVs of 5.5% and 2.6%, respectively. Chromogranin A (CgA) was measured by an immunochemiluminimetric assay with a sensitivity of 1.5 *μ*g/mL (Quest Diagnostics, San Juan Capistrano, CA). For metanephrines, normetanephrines, epinephrine, norepinephrine, and dopamine, we used enzymatic immunoassays (TriCat TM ELISA, IBL International GMBH, Germany). The same assays were used during the whole follow-up and repeated twice for quality control.

### 3.2. Pathology and Immunohistochemistry

The surgical specimen was fixed overnight in a phosphate-buffered, 10% formalin solution and later on, paraffin embedded and sliced. Routine hematoxylin-eosin (HE) staining was performed on 3 *μ*m sections of the paraffin blocks. Immunohistochemistry (IHC) was carried out on 2 *μ*m tissue sections using the following specific monoclonal antibodies (all purchased from DAKO Corp): corticotropin (ACTH, 1 : 50 dilution), chromogranin A (CgA, 1 : 50 dilution), synaptophysin (SNP, 1 : 50 dilution), PS-100 (1 : 400 dilution), CD56 (1 : 50 dilution), melan-A (1 : 50 dilution), calretinin (1 : 50 dilution), inhibin (1 : 50 dilution), p53 (1 : 50), steroidogenic factor-1 (SF-1, 1 : 50 dilution), and Ki67 (1 : 50 dilution). Double-labelling IHC was performed with two different chromogenes, horseradish peroxidase and alkaline phosphatase, each linked to monoclonal antibodies against CgA (to identify medullary cells) and inhibin (to identify cortical cells), respectively, using the same antibody dilutions and incubation times. All immunostains were standardized using appropriate controls.

### 3.3. SOX-2, NANOG, and OCT4 mRNA and Protein Expression

Total RNA was extracted and purified from frozen tumor tissue using the RNeasy tissue Mini Kit (Qiagen Inc., USA). After verifying integrity by means of Agilent Bioanalyzer 2100 (manufacturer), 1 *μ*g of purified RNA was retrotranscribed using the SuperScript VILO Master Mix (Applied Biosystems, CA, USA). The resulting cDNA was used to amplify SOX-2, NANOG, and OCT4 transcripts using specific forward and reverse primers, which are depicted in Table 2, along with the expected size of PCR products. End point PCR was carried out according to the following protocol: an initial, 5°minute, long denaturing step at 95°C followed by 40 cycles of 30 seconds at 95°C, 30 seconds at 60°C, and 30 seconds at 72°C, ending with a final 5°minute, final extension step. The gene encoding ribosomal protein subunit 18 (RPS18) was concomitantly amplified and used as an internal constitutive control. PCR products were visualized on 2%, ethidium bromide-stained agarose gel electrophoresis and their identity verified with the aid of molecular weight markers.

Immunofluorescence was performed on paraffin-embedded tumor tissues. They were first deparaffinized at 67°C by 30 min in xylene and subsequently hydrated with alcohol and distilled water. For epitope unmasking, the tissue sample was immersed in a 10 mM EDTA buffer at pH 8.05, autoclaved for 10 minutes, and washed with phosphate buffer (PBS) at pH 7.4. Autofluorescence was minimized by immersing the sample in 0.05 M NH4Cl for 30 minutes, and later on, cross sections were blocked with 3% bovine serum albumin (BSA) for 2 hours. The tissue sample was incubated with primary antibodies against SOX-2 (GTX627405-GeneTex), OCT-4 (GTX627419-GeneTex), and NANOG (GTX627421-GeneTex) diluted 1 : 100 in PBS containing 1% BSA for one hour and later on, with the Cy3-conjugated goat antibody against mouse IgG (81–6515, Invitrogen) for 30 minutes. Finally, the specimen was mounted on VECTASHIELD-DAPI mounting medium, and images were captured with a confocal microscope (Leica SP8, Barcelona, Spain).

The same assays were used for all the similar evaluations and repeated for quality control.

## 4. Results

### 4.1. Histopathology Evaluation

The excised tumor was a 330 g, 12 cm heterogeneous, tan-brown mass with bright yellow areas surrounded by a thin fibrous capsule (Figure 2(a)). The cut surface was soft and lobulated by fibrous septa with intermixed light gray-brown zones and granular necrotic mantles.

Histopathology of the tumor was complex. On HE, two different, yet, intermixed cellular patterns vaguely separated from each other were evident, designated from now on as patterns *A* and *C* with purely descriptive purposes (Figure 2(b)). Pattern *A* consisted of distinctly nested, medium-sized cells with scant to moderate cytoplasm, ill-defined cell membrane, oval nuclei with coarsely granular chromatin, and occasional inconspicuous nucleoli. Pattern *C* consisted of mantles and trabeculae of large polygonal cells with a well-defined cell membrane, abundant clear to eosinophilic granular cytoplasm, round to oval nuclei with small nucleoli, and finely dispersed chromatin. A third mixed pattern, pattern *B*, was observed in which the cells adopted to a variable degree, an intermediate or transitional phenotype between patterns *A* and *B*. Mitotic figures were numerous (up to 6 per high-power field) and randomly distributed. Mantles of ischemic necrosis constituted approximately 30% of the tumor volume.

### 4.2. Immunohistochemistry

Pattern *A* cells expressed a neuroendocrine immunophenotype as they positively stained for CgA, SNP, and CD56, as well as focally for ACTH. Interestingly, the cell nests of pattern *A* were rimmed by a discrete network of fusiform cells with slender cytoplasm and elongated nuclei with dense chromatin that immunostained for PS-100. Pattern *C* cells expressed an adrenocortical phenotype characterized by positive immunostaining for SF-1, inhibin, calretinin, and melan-A. Immunohistochemical studies confirmed our impression upon H&E staining that the two cellular patterns were intermingled, without clear limits between them, and frequently exhibiting transitional areas (i.e., pattern *B*). Proliferative index assessed by Ki-67 immunostaining was 40%, preferentially expressed by pattern “C” cell type, although not restricted to it. Double-labelling IHC confirmed the coexistence of medullary (anti-CgA antibodies) and cortical (anti-inhibin antibodies) cells within the tumor, with the former somewhat predominating over the latter (Figures 2(c) and 2(d)). Interestingly, a few of the cells clearly showed reactivity to both antibodies.

### 4.3. SOX-2, NANOG, and OCT4 mRNA and Protein Expression

**Figure 3:**
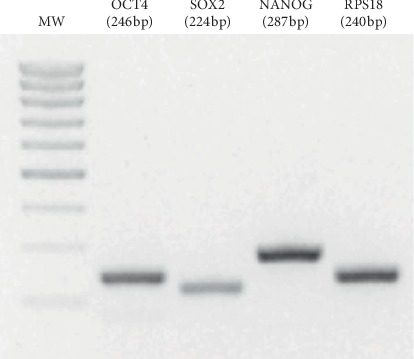
Ethidium bromide-stained agarose gel electrophoresis of RT/PCR products showing clearly defined bands corresponding to NANOG, OCT4, and SOX_2_ amplicons of the expected size (RPS18: ribosomal protein S18).

RT/PCR using specific primers for SOX-1, NANOG, and OCT4 confirmed the expression of the corresponding transcripts of the expected molecular weight as shown in Figure 3.

**Figure 4:**
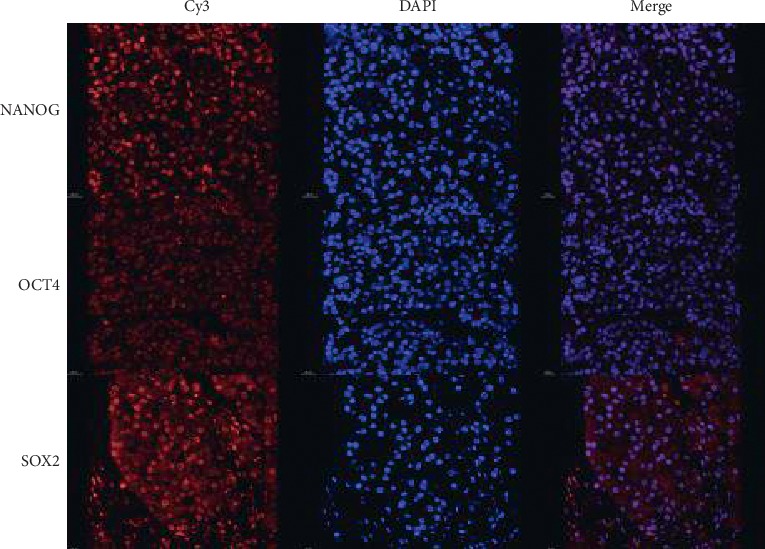
Immunofluorescence staining showing NANOG nuclear reactivity; OCT4 reactivity is predominantly nuclear and scant in the cytoplasm; SOX_2_ immunoreactivity is localized to both nucleus and cytoplasm (Cy3: cyanine dye; DAPI: 4',6-diamidino-2-fenilindol).

Immunofluorescence using specific antibodies against stem cell antigens revealed predominantly nuclear NANOG and OCT4 reactivity, whereas SOX_2_ immunoreactivity was localized to both the nucleus and the cytoplasm (Figure 4).

## 5. Discussion

The differential diagnosis of Cushing's syndrome represents one of the major challenges that clinical endocrinologists face. A sequential work up, usually starting with screening tests aimed to confirming the existence of endogenous hypercortisolism followed by the establishment of ACTH dependency or independency and ending with biochemical tests and imaging techniques aimed at localizing the original source of the condition, is essential for an accurate diagnosis and thus for the establishment of an effective therapeutic plan [11]. In the vast majority of cases, adrenal Cushing's syndrome is due to cortical adenomas and more rarely carcinomas, which are ACTH-independent conditions [12, 13]. Patients with ACTH-dependent Cushing's syndrome have either a pituitary ACTH-producing adenoma (Cushing's disease) or ectopic ACTH production associated to a neuroendocrine neoplasm that is more commonly located in the thymus, lungs, or endocrine pancreas [11, 13]. Over 80% of patients with Cushing's syndrome have an ACTH-secreting corticotroph adenoma, whereas ectopic 30 ACTH syndrome (EAS) accounts for 20% of all adult cases [11, 13]. Overall, EAS is very rare and has an incidence of 1–4 new cases per 10 million inhabitants per year and is extremely rare in young or pediatric populations [14, 15].

We present a very young patient, with severe clinical and biochemical ACTH-dependent Cushing's syndrome. The lack of cortisol suppression with the administration of 8 mg of DXM, the absence of a pituitary adenoma upon MRI, and the lack of a petrosal-to peripheral ACTH gradient pointed to an ectopic ACTH source. CT scanning of the chest and abdomen did not reveal any bronchial, thymic, or pancreatic lesions; instead and somewhat to our surprise given the patient's ACTH levels, we found a large heterogeneous right adrenal mass. Although urinary metanephrines and catecholamines were within normal limits and the adrenal mass did not take up MIBG, we entertained the possibility of a pheochromocytoma as the actual source of ectopic ACTH, being the only previous adrenal EAS-related tumors reported so far. The patient underwent surgical resection of the adrenal mass, which proved to be a mixed corticomedullary tumor, consisting of two distinct but intermingled populations of cells: the classical medullary cells with “zellballen” appearance arranged in nests, surrounded by sustentacular cells, and an adrenocortical component with a different phenotype and a third population showing both characteristics. It must be pointed out that our patient harbored a single primary tumor with different cell populations and not different tumors that grew enough to collide with each other, also called colliding tumors, which may be found in all sorts of publications regarding mixed tumors, and that may have different origins, pathophysiology, and outcomes. As in previously reported cases of truly MCAT tumors, the medullary component immunostained positively for the usual chromaffin markers (SNP, CgA, S-100, and CD56), and it had a clearly positive ACTH immunohistochemistry (which is a requirement to diagnose ectopic sources of ACTH). The adrenocortical carcinoma component was positive for epithelial markers such as melan-A, inhibin, and calretinin with a very high Ki-67 proliferative index of 40%, which reflects the malignant potential of the lesion. Most neuroendocrine neoplasias are well-differentiated tumors that usually report Ki-67 immunostainings of <2% and very rarely show Ki-67% over 20%. High Ki-67 and a poorly differentiated morphology classify the tumor as a grade 3 neuroendocrine carcinoma, which predicts a poor prognosis right from the beginning. On the contrary, Ki-67 values in adrenal carcinomas are quite variable, ranging from 2 to 80%, but a mitotic index of >10% is considered to be of poor prognosis factor. The proper cutoff for mitotic indexes for all endocrine tumors is still a matter of debate, but by any definition, this tumor showed an atypical behavior that could not be ignored and warranted close follow-up with laboratory and imaging even if the patient had no clinical signs of tumor recurrence. We should clarify that the patient showed biochemical recurrence a few weeks before the clinical symptoms recurred. There were no clear signs of tumor recurrence in the previous imaging study despite the frequency with which they were done, stating the fast growth and aggressiveness of the metastasis. This should remind all the interdisciplinary team members that we need more than one test to detect tumor growth and that full-blown clinical syndromes may be related to advanced disease.

We considered the possibility of the presence of a stem cell origin due to the atypical tumor pathology and behavior as well as the reports of higher frequencies of stem cell tumors in the pediatric population. The adrenocortical niche is complex, and some aspects of its development have been studied recently. Alterations in these mechanisms are associated with pediatric adrenal tumors, including stem cells. Pediatric adrenal tumors are usually detected in the first few years of life, but this patient would still be considered as a teenager or a young adult. She may be old for the classical pediatric tumor, but she is considerably young for the classical adult presentation which is over 50 years of age. Guidelines for many neoplasias, including endocrine neoplasias, consider the existence of “atypical tumors,” which may warrant further suspicion and evaluations. They may be defined as atypical if they are large, multihormonal, multicentric, or without classical risk factors for their presentation. The definition may change for every organ and pathology. This definition includes most mixed pathology tumors.

Mixed corticomedullary tumors are extremely rare, including the present one, and there are only 26 cases reported in the English literature since its initial description in 1969 by Mathison et al. [16]. However, the medullary component has usually been reported as a benign pheochromocytoma [17], and the adrenocortical component can be either an adenoma [1–3, 6, 7, 9] or a carcinoma [4, 5, 8]. There have been only 3 previously reported cases of malignant, mixed adrenocortical tumors [4, 5, 8]. In one of these cases [4], the medullary and cortical tum or cells appeared to collide with each other, whereas in the other two patients [5, 8], like in our case, the two cell populations do not collide but are intermingled instead. Interestingly, none of these three patients presented with clinical signs or symptoms of hypercortisolism, in contrast to our patient who had severe clinical and biochemical, ACTH-dependent Cushing syndrome.

The coexistence of cortical and medullary cells was demonstrated by double-labeling IHC using antibodies against CgA and inhibin. Interestingly, a few of these cells positively immunostained for both markers, which prompted us to hypothesize that this mixed tumor may have arisen from a common pluripotential stem cell. After confirming that the tumor expressed its mRNAs by means of RT/PCR, we performed immunofluorescence studies using antibodies against OCT4, NANOG, and SOX2. The tumor immunostained for these three antigens which are considered to be cancer stem cell biomarkers, since they are critical regulators of self-renewal and pluripotency of embryonic stem cells, and thus can regulate proliferation and differentiation [18, 19]. OCT4 is a POU domain transcription factor that promotes epithelial-mesenchymal transition and inhibits apoptosis through the regulation of miR125B-BAK1 pathways [19]. SOX_2_ is involved in the control of cell proliferation, apoptosis, invasion, migration, and metastases in various cancers by promoting epithelial-mesenchymal transition, upregulating WNT/beta-catenin, and downregulating AMPK/mTOR pathways [18]. NANOG contributes to carcinogenesis by activating and preserving cancer stem cell properties; it regulates signaling pathways such as STAT3/Snail [20, 21]. OCT4 has a characteristic nuclear staining pattern specific for germ cell neoplasms [22]. Cytoplasmic expression of OCT4 has been described in metastatic pheochromocytomas and in moderately differentiated neuroendocrine tumors [23] but has not been reported before in adrenocortical carcinomas.

ACTH is known to be synthesized by the chromaffin cells of the adrenal medulla and to participate in complex intraglandular autocrine and paracrine loops regulating steroidogenesis in the adrenal cortex [24]. Although exceedingly rare, pheochromocytomas have been known to be a potential source of ectopic ACTH, and only 58 such cases have been reported in the English medical literature between 1977 and 2018 [10, 17]. In most of these patients, the contralateral adrenal gland looks grossly hyperplastic on imaging studies and is most likely the origin of the excessive cortisol secretion; however, contralateral atrophy has also been reported in pheochromocytomas with ectopic production of ACTH. Although many case reports and series do not always make a point of mentioning this, some published cases show similar behavior to that of our patient.

Interestingly, several recent reports of mixed corticomedullary tumors describe the tomographic characteristics of the affected gland but omit describing the state of the contralateral adrenal [7, 8, 17]. However, a direct comparison with other mixed tumors is not possible since the other case reports did not undergo dynamic testing to prove an ectopic ACTH source.

The true reason why the contralateral gland is not enlarged despite the presence of an ectopic source of ACTH cannot be proven at this point. The authors have considered some hypotheses in this regard:

1. Some of the circulating ACTH may correspond to fragments or inactive ACTH, which has been reported as a common consequence of the faulty mRNA processing and peptide secretory mechanisms of these tumors. These isoforms cannot be detected by our assay.
2. The glucocorticoid excess may have been present for a longer time, but it may have been insidious or “subclinical” for some weeks or months before the aggressive clinical presentation was detected. Since adrenal glands can change their morphology rapidly (in the course of days to weeks) according to the cholesterol deposits, their metabolic activity and the present stimuli, even a few weeks of hypercortisolism, may cause atrophy in some glands.
3. The atrophic adrenal gland may be indicative of a persistent and obvious effect of systemic hypercortisolism, while the nonsuppressed ACTH can be related to ACTH dependence, but not necessarily to its trophic effects. We could expect to see less local effects of the ACTH in the smaller gland if the excessive cortisol signals (atrophy) were stronger than the ACTH signals (hypertrophy).
4. One atrophic gland is also suggestive of the fact that we were not dealing with a case of bilateral macronodular adrenal hyperplasia.

We should also comment that postoperative ACTH was not performed immediately after surgery, but a few weeks later. Immediate ACTH determination after surgery was not considered to be useful at that point due to the fact that the patient was in critical conditions after surgery and that an appropriate elevation of ACTH in response to stress and a sudden decrease of blood cortisol is expected during the first few days when a normal pituitary gland is present. It is also not possible to distinguish the ectopic ACTH from the pituitary ACTH using our assay. A normal or elevated ACTH was also to be expected after the patient was determined to be hypocortisolic in the postoperative evaluations and treatment with prednisone was started. After the morning serum cortisol came back to normal, prednisone was withdrawn, and then we determined ACTH again, which came back normal or slightly elevated, until the presence of the liver metastasis, when it became suppressed.

Despite having received radiation therapy, but to some extent, expectedly, Cushing's syndrome recurred 16 months after surgery, along with the appearance of new hepatic lesions, likely corresponding to metastases. Although the primary tumor was clearly an ACTH-dependent lesion, such dependency was lost once it became metastatic. It was decided not to biopsy these liver lesions because at this point, the patient was receiving only palliative care. Nevertheless, we concluded based on the patient's hormonal profile that they were hormonally active, secreting cortisol and perhaps androgens in an autonomous, ACTH-independent fashion. Supporting the latter is the fact that the contralateral gland looked somewhat hypoplastic on imaging studies. It is interesting to speculate that the medullary component of this mixed tumor seems to require the adrenal microenvironment to produce a biologically active ACTH that would act in a paracrine/autocrine fashion, stimulating steroidogenesis. We believe that the ectopic ACTH produced by the mixed adrenal tumor acted mostly inside the same adrenal gland in a paracrine manner. The affected gland was enlarged and was the only one producing adrenal steroids, while the other one was suppressed. This suppression could explain the atrophy seen in the contralateral adrenal gland and the transient hypocortisolism seen after adrenalectomy. It also supports the idea that the liver metastases were functioning and ACTH independent. We did not find any other MCAT case reports where ectopic ACTH dependency was completely ruled out by a prolactin-controlled petrous sinus sampling and 3 Tesla, contrasted, and dynamic MRI imaging, but we found some reports where ACTH was abnormally normal (nonsuppressed) or high. These tumors might also have ectopic ACTH production, but they were not tested for it. According to several guidelines, complete hormonal workup is mandatory for all adrenal tumors suspicious for malignancy; screening studies should be followed by appropriate endocrine dynamic tests when necessary. Despite the presence of endocrine dysfunction and the worse outcomes reported for functioning tumors, specialized testing is still underused by many specialists. It has also been shown that a thorough evaluation of atypical cases helps to enlighten the pathophysiology of more common cases, although more cases need to replicate these findings in order to study other hypotheses.

In conclusion, we have described the case of a young woman with a MCAT, consisting of cells with pheochromocytoma and adrenocortical carcinoma features. We have identified by double-labeling immunohistochemistry the presence of a few cells within the tumor that immunostained for both cortical and medullary antigens, which together with the positive immunofluorescence for cancer stem cell biomarkers (OCT4, NANOG, and SOX-2) points towards the involvement of primitive embryonic cells as the origin of this neoplasm.

## Figures and Tables

**Table 1:** Hormonal evaluation at diagnosis and at 3 and 16 months after right adrenalectomy.

	At diagnosis	3 months PO	16 months PO	Normal range
LH (mIU/mL)	<0.1	13.47	0.9	2–7
FSH (mIU/mL)	<0.1	5.16	<0.1	2–7
Estradiol (pg/mL)	62.5	16.52	10	50–200
Total testosterone (*μ*g/dL)	42.4	19.25	24.3	<40
DHEA-s (*μ*g/dL)	379	25.6	77.2	61–493
ACTH (pg/mL)	38.6	12.27	2.35	10–50
Urinary free cortisol (*μ*g/day)	2798	20.23	849	12.8–82.5
Aldosterone (pg/mL)	140.3	—	—	12–157
PRA (ng/mL/h)	17.1	—	—	0.2–2.8
CgA, *x* upper limit of normal	1.26	0.33	—	1

**Table 2:** Primer sequences and expected PCR product sizes.

	Sequence	Product size (bp)
SOX2 forward	ACACCAATCCCATCCACACT	224
SOX2 reverse	GCAAACTTCCTGCAAAGCTC	
NANOG forward	TGCAAATGTCTTCTGCTGAGAT	287
NANOG reverse	GTTCAGGATGTTGGAGAGTTC	
OCT4 forward	CGTGAAGCTGGAGAAGGAGAAGCTG	246
OCT4 reverse	AAGGGCCGCAGCTTACACATGTTC	
RPS18 forward	AATCCACGCCAGTACAAGATCCCA	240
RPS18 reverse	TTTCTTCTTGGACACACCCACGGT	

## Abbreviation

ACTH: Adrenocorticotropic hormone
AMPK: Activated protein kinase
CgA: Chromogranin A, biomarker for neuroendocrine cells
CT Scan: Computed tomography scan
DHEA-s: Dehydroepiandrosterone sulfate
EAS: Ectopic ACTH syndrome
FSH: Follicle-stimulating hormone
HE or H&E: Hematoxylin eosin
IHC: Immunohistochemistry
LH: Luteinizing hormone
MCAT: Mixed corticomedullary adrenal tumors
MIBG: Metaiodobenzylguanidine
MRI: Magnetic resonance imaging
mRNA: Messenger ribonucleic acid
mTOR: Mammalian target of rapamycin
NANOG: Tír Na nÓg, an homeobox transcription factor named after the Irish legend of Oisín
OCT4: Octamer-binding transcription factor 4
PRA: Plasmatic renin activity
PS-100: Protein S 100, biomarker for a nerve sheet origin
RT/PCR: Real-time polymerase chain reaction
SN: Synaptophysin
SOX_2_: Transcription factor sex-determining region *Y*-box 2
STAT3: Signal transducer and activator of transcription 3
UFC: Urinary free cortisol
WNT: Portmanteau of the wingless and int genes, stands for wingless-related integration site
99Tc-HYNIC: 99metastableTechnetium-ethylenodiamine–N-N' diacetic acid-HYNIC-TOC [D-Phe1, Tyr3-Octreotide].
