# Erratum to “From ACTH-Dependent to ACTH-Independent Cushing's Syndrome from a Malignant Mixed Corticomedullary Adrenal Tumor: Potential Role of Embryonic Stem Cells”

**DOI:** 10.1155/2020/1704902

**Published:** 2020-09-09

**Authors:** Claudia Ramirez-Rentería, Ana-Laura Espinosa-de-los-Monteros, Etual Espinosa-Cardenas, Daniel Marrero-Rodriguez, Guillermo Castellanos, Rocio Arreola-Rosales, Guillermo Montoya-Martinez, Keiko Taniguchi-Ponciano, Paola Briseño-Diaz, Mayte-Lizeth Padilla-Cristerna, Maria-del-Pilar Figueroa-Corona, Moises Mercado

**Affiliations:** ^1^Endocrine Research Unit, Hospital de Especialidades, CMN S.XXI, IMSS, Mexico City, Mexico; ^2^Endocrinology Service, Hospital de Especialidades, CMN S.XXI, IMSS, Mexico City, Mexico; ^3^Pathology Department, Hospital de Especialidades, CMN S.XXI, IMSS, Mexico City, Mexico; ^4^Urology Service, Hospital de Especialidades, CMN S.XXI, IMSS, Mexico City, Mexico; ^5^Department of Molecular Biomedicine, Centro de Estudios Avanzados, Instituto Politécnico Nacional, Mexico City, Mexico

In the article titled “From ACTH-Dependent to ACTH-Independent Cushing's Syndrome from a Malignant Mixed Corticomedullary Adrenal Tumor: Potential Role of Embryonic Stem Cells” [1], there was a spelling error in the author's name in the author list, where Mercado Moises should have read as Moises Mercado. The revised authors' list is shown above.
